# Empathy ability of nursing students: A systematic review and meta-analysis

**DOI:** 10.1097/MD.0000000000030017

**Published:** 2022-08-12

**Authors:** Jiao Jia-Ru, Zheng Yan-Xue, Hao Wen-Nv

**Affiliations:** a School of Nursing, Inner Mongolia Medical University, Hohhot, Inner Mongolia, China; b Department of Emergency, Affiliated Hospital of Inner Mongolia Medical University, Huimin District, Hohhot, Inner Mongolia, China.

**Keywords:** empathy, empathy ability, meta-analysis, nursing students, undergraduates

## Abstract

**Background:**

Empathy is an ability that nursing students need in clinical practice, there is no available data to assess nursing students’ empathy ability level . The main purpose of this study is to synthesize the evidence relating to the empathy ability in nursing students to systematically evaluate the empathy ability level among nursing students.

**Methods:**

Adhering to the preferred reporting items for Systematic Reviews and Meta-analyses guidelines, we searched PubMed, Cochrane, Web of Science, Scopus, ScienceDirect, Wiley Library, Embase, CNKI, Wanfang, and China biomedical literature service system ten databases to collect cross-sectional studies on nursing students’ empathy ability. Two researchers independently screened the literature, extracted the data, and evaluated the risk of bias in the included studies.

**Results:**

A total of 19 cross-sectional studies were included. The sample comprised 5407 nursing students. Meta-analysis showed that females have a higher empathy ability than males, and the empathy ability of rural students is higher than that of provincial students.

**Conclusions:**

The findings suggest that the empathy ability among nursing students worldwide is higher, but there needs further improvement. This result makes nursing educators pay more attention to the cultivation of the nursing students’ empathy ability; improving the empathy level is beneficial in improving the standards of health care and patients’ quality of life.

## 1. Introduction

Empathy is often described as the feeling that a person image themselves in another’s situation and “putting himself in the other’s shoes.” It represents the skill of understanding other people’s feelings and meaning, then communicating those feelings to others.^[1]^ Empathy is an aspect of personality that plays an important role in interpersonal relationships and promoting communication skills.^[2]^ Empathy is a prerequisite for effective nursing and a comprehensive understanding of the patient’s viewpoint.^[3]^

In nursing and medical practice, high levels of empathy benefit patient health and clinical outcomes, such as reduced psychological stress, improved self-concept, reduced anxiety and depression, and lower complication rates.^[3–5]^ In addition, compassion and empathy play an important role in providing quality care, and they are important for nursing students and the nursing profession to develop.^[6]^

However, there are few studies on the empathy of nursing students. Previous studies mainly focused on empathy levels among nurses, empathy ability differences among health professionals, and the relationship between empathy and participant variables.^[7]^ It has been understood that nursing students need to acquire not only technical skills but also human and relationship skills.^[8]^ So, nursing trainers and teachers should start with basic education to develop the empathy ability in nursing students and maintain it at a high level.^[9]^

Therefore, it is necessary to know the level of empathy ability of nursing students to adjust the training program. No pooled data is available to assess the level of empathy ability among nursing students. Hence, this meta-analysis is conducted. Results of this study can help nursing educators to understand the overall level and influencing factors of empathy of nursing students and adjust learning training programs to improve their empathy ability.

## 2. Materials and Methods

### 2.1. Data sources

**Figure 1.**
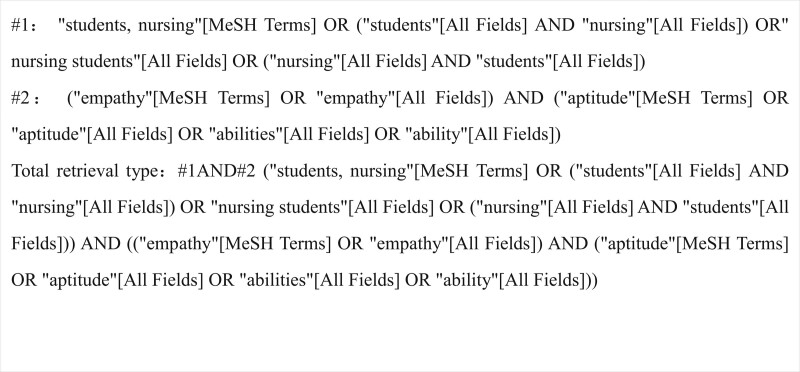
Search strategy.

The following academic databases were searched from the establishment of the database to October 2021. We searched PubMed, Cochrane, Web of Science, Scopus, ScienceDirect, Wiley Library, Embase, CNKI, Wanfang, and China biomedical literature service system databases to collect cross-sectional studies on nursing students’ empathy ability. All the retrieval methods are based on the combination of subject and free words and are adjusted according to the specific database. The retrieval strategy is determined after multiple preretrieval. English keywords include empathy ability, nursing students, etc. Taking PubMed as an example, the specific search strategy is shown in Figure 1.

### 2.2. Eligibility criteria

Apply the following eligibility criteria in selecting appropriate studies for analysis: the subjects were nursing undergraduates or a mixed sample of nursing undergraduates and junior college students; the study design was cross-sectional, and they should be primary and quantitative; at least one of the research indicators was measured by standardized and validated instruments; based on a sample of nursing undergraduates or on a mixed sample, the results for nursing undergraduates are provided separately; published in the English or Chinese language; and peer-reviewed studies are available in full text.

### 2.3. Study selection and data extraction

Two researchers (J.J.-R and Z.Y.-X) independently screened literature, extracted data, and cross-checked. In case of disagreement, it shall be settled through discussion or negotiation with the third party (H.W.-N.). When selecting the articles, first read the title. After excluding the unrelated articles, further read the abstract and full text to determine whether they are included. Data extraction includes basic information about the included studies: first author, year of publication, survey period, total sample size and source region, etc; outcome indicators: mean and standard deviation of empathy ability score for nursing students; and the related elements of bias risk assessment.

### 2.4. Statistical analysis

Endnote X9 was used to summarize the articles. Excel software was used for data extraction management, statistics, and descriptive analysis of outcome indicators. RevMan 5.4 software was used for meta-analysis. The continuous variables are represented by standardized mean difference (SMD) and 95% confidence interval (95% CI). The chi-square test and *I*² index were used to determine whether there was heterogeneity among studies, and the heterogeneity of effect sizes was analyzed. If there was no heterogeneity among studies (*P* > .1, *I*² < 50%), the fixed-effect model was adopted. If there was heterogeneity among studies (*P* < .1, *I*² ≥ 50%), the random-effects model was used to combine effect sizes.

### 2.5. Quality appraisal

Two reviewers (J.J.-R. and Z.Y.-X.) in the form of mutual blindness independently evaluated the included literature using the Agency for Health Care Research and Quality tool.^[10]^ The Agency for Health Care Research and Quality tool mainly consists of 11 items. If the answer is “no” or “unclear,” the item score is “0”; If the answer is “yes,” the item score is “1.” A score of 8 to 11 is considered high quality, 4 to 7 moderate quality, and <4 poor quality. After the independent evaluation, 2 researchers will discuss and reach a consensus. If there is any disagreement, the third researcher (H.W.-N.) will arbitrate, or the research group will discuss and decide.

### 2.6. Ethical consideration

Ethical approval was not required based on the use of already published secondary data and the meta-analysis nature.

## 3. Results

### 3.1. Literature screening process and results

**Figure 2.**
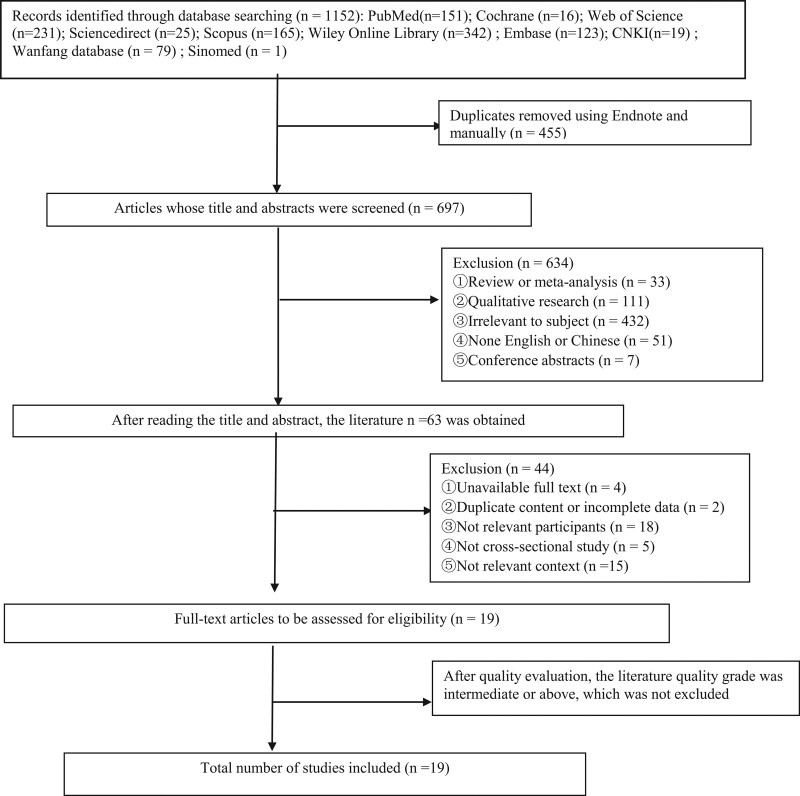
Literature screening process and results.

A total of 1152 articles were identified, 455 duplicate articles were removed, leaving 697 papers for further screening. Subsequently, 2 reviewers read titles and abstracts to eliminate 634 unqualified articles in non-English or Chinese, conference abstracts, qualitative studies, reviews or meta-analyses, and irrelevant to the topic. In total, 63 articles were included for full-text review. From these, 44 unqualified articles were eliminated, such as unable to obtain full text, duplicate content or incomplete data, inconsistent research object and content, and non–cross-sectional research type. Finally, 19 studies^[11–29]^ met the inclusion criteria as shown in Figure 2.

### 3.2. Basic characteristics of included studies

The 19 articles used cross-sectional studies, and all quantitative studies used validated scales for data collection. The included literature was published from 2010 to 2022. The sample size of the study subjects was 502 at most and 106 at least, and the findings of these studies are based on a total of 5407 participants. The basic characteristics of the included studies are shown in Table 1.

**Table 1:** Basic characteristics of included studies.

Author, year, country	Subjects	Design	Date collection	Gender	Place of birth	Overall average score
Öztürk and Kaçan, 2022, Turkey^[11]^	Total: n = 430	Descriptive study	Empathic Skill Scale (ESS)	--Female		145.97 ± 23.57
	-Female: n = 249			150.02 ± 24.10		
	-Male: n = 181			--Male:		
				140.39 ± 21.67		
Kaplan and Tülüce, 2021, Turkey^[12]^	Total: n = 229	Descriptive or cross-sectional study	Empathic Tendency Scale	--Female:	--Province:	68.78 ± 8.88
	-Female: n = 178			69.03 ± 8.43	69.06 ± 9.46	
	-Male: n = 51			--Male:	--District:	
	-Province: n = 94			67.98 ± 10.19	69.48 ± 8.70	
	-District: n = 99				--Village:	
	-Village: n = 36				66.25 ± 7.04	
Oh, 2019, South Korea^[13]^	Total: n = 247	Descriptive quantitative study	Korean version of the Interpersonal Reactivity Index (IRI)			3.56 ± 0.39
Petrucci et al, 2016, Italy^[14]^	Total: n = 502	Comparative cross-sectional study	Italian-validated version of the Jefferson Scale of Empathy	--Female:		113.52 ± 11.56
	-Female: n = 333			113.82 ± 12.71		
	-Male: n = 169			--Male:		
				106.92 ± 12.11		
Elizabeth et al, 2021, Colombia^[15]^	Total: n = 253	Exploratory and cross-sectional study	Jefferson Medical Scale of Empathy (Version-S)	--Female:		95.07 ± 20.647
	-Female: n = 123			100.65 ± 21.881		
	-Male: n = 130			--Male:		
				89.78 ± 17.949		
Jakob et al, 2019, Sweden^[16]^	Total: n = 329	Comparative cross-sectional study	Jefferson Scale of Physician Empathy (JSPE)			The sixth semester:
						117.29 ± 11.32
						The second semester:
McKenna et al, 2012, Australia^[17]^	Total: n = 106	Cross-sectional study	Jefferson Scale of Physician Empathy (JSPE)			107.34 ± 13.74
Zhu et al, 2016, China^[18]^	Total: n = 344	Convenience sampling	Jefferson Scale of Empathy for Nursing Students (JSPE-NS)	-Female:	-Province:	104.71 ± 15.543
	-Female: n = 327			104.99 ± 15.481	106.35 ± 16.635	
	-Male: n = 17			-Male:	-Village:	
	-Province: n = 136			99.24 ± 16.204	103.63 ± 14.727	
	-Village: n = 208					
Kang 2013, China^[19]^	Total: n = 299	Stratified sampling	Interpersonal Reactivity Index			52.68 ± 10.53
Liu et al, 2016, China^[20]^	Total: n = 220	Cluster random sampling	Jefferson Scale of Empathy for Nursing Students (JSPE-NS)			111.44 ± 7.13
Li, 2017, China^[21]^	Total: n = 402	Cluster random sampling	Interpersonal Reactivity Index Chinese Version (IRI-C)		-Province:	53.52 ± 8.46
	-Province: n = 158				52.37 ± 8.67	
	-Village: n = 244				-Village:	
					54.27 ± 8.25	
Guo et al, 2020, China^[22]^	Total: n = 262	Cross-sectional study	Jefferson Scale of Empathy for Nursing Students (JSPE-NS)	-Female:	-Province:	108.12 ± 14.69
	-Female: n = 223			109.32 ± 14.40	109.06 ± 14.98	
	-Male: n = 39			-Male:	-Village:	
	-Province: n = 88			101.30 ± 14.70	107.65 ± 14.56	
	-Village: n = 174					
Wang, 2010, China^[23]^	Total: n = 184	Descriptive study	Interpersonal Reactivity Index Chinese Version (IRI-C)	-Female:	-Province:	57.05 ± 7.84
	-Female: n = 174			57.44 ± 7.55	57.95 ± 8.60	
	-Male: n = 10			-Male:	-Village:	
	-Province: n = 38			50.30 ± 9.98	56.82 ± 7.64	
	-Village: n = 146					
Li et al, 2012, China^[24]^	Total: n = 351	Stratification facilitates cluster sampling	College Student Empathy Scale			99.94 ± 12.06
Xu et al, 2020, China^[25]^	Total: n = 118	Chester sampling	Jefferson Scale of Empathy for Nursing Students (JSPE-NS)	-Female:	-Province:	104.96 ± 13.26
	-Female: n = 100			105.25 ± 12.86	105.92 ± 12.62	
	-Male: n = 18			-Male:	-Village:	
	-Province: n = 26			103.33 ± 15.64	104.68 ± 13.49	
	-Village: n = 92					
Zheng et al, 2020, China^[26]^	Total: n = 472	Convenience sampling	Jefferson Scale of Empathy-Health Professionals (JSE-HP)	-Female:	-Province:	105.24 ± 12.00
	-Female: n = 445			105.87 ± 11.49	103.53 ± 13.65	
	-Male: n = 27			-Male:	-Village:	
	-Province: n = 111			94.70 ± 15.26	105.76 ± 11.42	
	-Village: n = 361					
Ge et al, 2020, China^[27]^	Total: n = 300	Convenience sampling	Jefferson Scale of Empathy for Nursing Students (JSPE-NS)	-Female:		108.74 ± 12.44
	-Female: n = 275			109.00 ± 12.20		
	-Male: n = 25			-Male:		
				105.90 ± 14.05		
Lu and Chen, 2018, China^[28]^	Total: n = 209	Questionnaire survey	Jefferson Empathy Scale in Chinese	-Female:	-Province:	87.60 ± 16.40
	-Female: n = 189			87.90 ± 16.10	85.40 ± 15.80	
	-Male: n = 20			-Male:	-Village:	
	-Province: n = 113			88.70 ± 16.40	86.20 ± 14.60	
	-Village: n = 96					
Yang et al, 2019, China^[29]^	Total: n = 150	Questionnaire survey	Interpersonal Reactivity Index Chinese Version (IRI-C)	-Female:		51.67 ± 9.40
	-Female: n = 133			53.79 ± 9.79		
	-Male: n = 17			-Male:		
				54.94 ± 12.27		

### 3.3. Basic risk assessment results of included studies

The bias risk assessment results of the included studies are shown in Table 2. Among the 19 articles, the quality assessment grade of 4 studies was high and that of 15 was medium.

**Table 2:** Bias risk assessment results of included studies.

Studies	②①	③②	④③	⑤④	⑥⑤	⑦⑥	⑧⑦	⑨⑧	⑩⑨	⑪⑩	⑪	Score	Grade
Öztürk and Kaçan^[11]^	Y	Y	N	Y	Y	N	N	Y	N	Y	N	6	Medium
Kaplan and Tülüce^[12]^	Y	Y	N	Y	Y	N	N	Y	N	Y	N	6	Medium
Oh^[13]^	Y	Y	Y	N	Y	N	Y	Y	Y	Y	N	8	High
Petrucci et al^[14]^	Y	Y	N	Y	Y	N	N	Y	N	Y	N	6	Medium
Elizabeth et al^[15]^	Y	Y	Y	Y	Y	N	N	Y	N	Y	N	7	Medium
Jakob et al^[16]^	Y	Y	N	Y	Y	N	N	Y	N	Y	N	6	Medium
McKenna et al^[17]^	Y	Y	N	N	Y	N	Y	Y	Y	Y	N	7	Medium
Kang^[19]^	Y	Y	N	N	Y	Y	Y	Y	Y	Y	N	8	High
Liu et al^[20]^	Y	Y	N	N	Y	N	Y	Y	Y	Y	N	7	Medium
Li^[21]^	Y	Y	N	N	Y	N	Y	Y	Y	Y	N	7	Medium
Guo^[22]^	Y	Y	N	N	Y	N	Y	Y	Y	Y	N	7	Medium
Wang^[23]^	Y	Y	N	N	Y	N	Y	Y	Y	Y	N	7	Medium
Li et al^[24]^	Y	Y	N	N	Y	N	Y	Y	Y	Y	N	7	Medium
Xu et al^[25]^	Y	Y	Y	Y	Y	N	Y	Y	N	Y	N	8	High
Zhu et al^[18]^	Y	Y	N	N	Y	N	Y	Y	Y	Y	N	7	Medium
Zheng et al^[26]^	Y	Y	N	Y	Y	N	N	Y	N	Y	N	6	Medium
Ge et al^[27]^	Y	Y	N	N	Y	N	Y	Y	Y	Y	N	7	Medium
Lu and Chen^[28]^	Y	Y	Y	Y	Y	N	N	Y	N	Y	N	7	Medium
Yang et al^[29]^	Y	Y	Y	N	Y	N	Y	Y	Y	Y	N	8	High

### 3.4. Meta-analysis results

#### 3.4..1. Global empathy ability.

**Figure 3.**
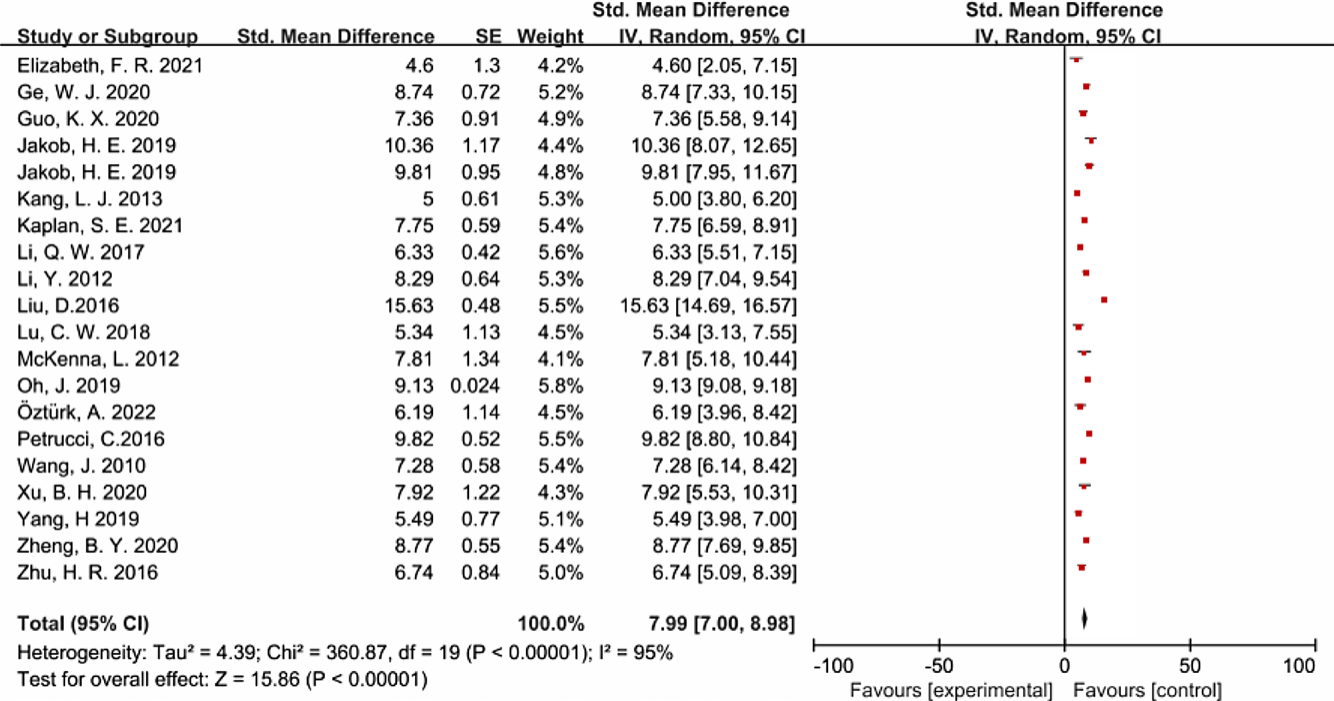
A meta-analysis of global empathy ability. CI = confidence interval.

The global empathy ability among nursing students was SMD = 7.99 (95% CI 7.00–8.98) with significant heterogeneity across the studies (χ² = 4.39; *df* = 19, *P* < .00001; *I*² = 95.0%). This global empathy ability was yielded based on all 19 studies and is demonstrated by the forest plot in Figure 3.

#### 3.4..2. Subgroup analyses.

**Figure 4.**
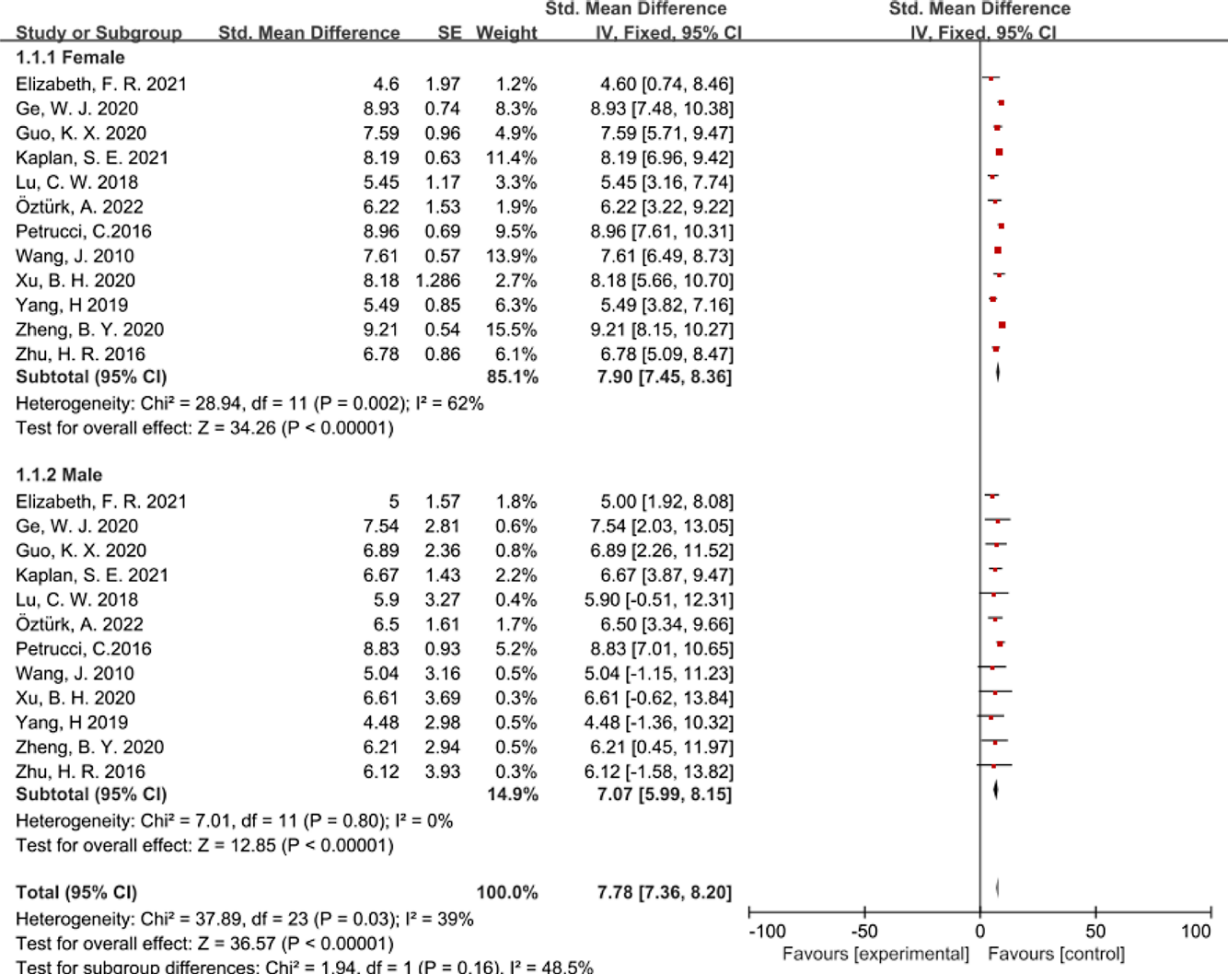
Forest plot assessing empathy ability among nursing students, stratified by gender of participants. CI = confidence interval, SE = standard error.

Analyses were conducted across the groups of gender of participants in 12 studies^[11,12,14,15,18,22,23,25–29]^ (Fig. 4). Females have a higher ability for empathy than males at SMD of 7.90 (95% CI 7.45–8.36). The differences between the subgroups were statistically insignificant (χ^2^ = 1.94, *df* = 1, *P* = .16, *I*² = 48.5%).

**Figure 5.**
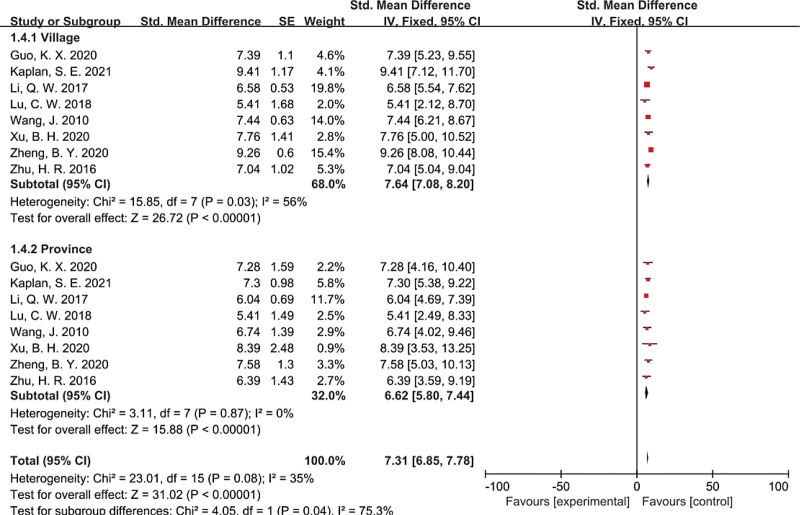
Forest plot assessing empathy ability among nursing students, stratified by place of birth for participants. CI = confidence interval, SE = standard error.

Analyses were conducted across the groups of places of birth for participants in 8 studies^[12,18,21–23,25,26,28]^ (Fig. 5). The empathy ability of nursing students born in provincial areas was at SMD of 6.62 (95% CI 5.80–7.44). The empathy ability of nursing students born in village areas was at SMD of 7.64 (95% CI 7.08–8.20). And the empathy ability of rural students is higher than that of provincial students. The differences between the subgroups were statistically significant (χ^2^ = 4.05, *df* = 1, *P* = .04, *I*² = 75.3%).

## 4. Discussion

### 4.1. Global empathy ability

As the first systematic review and meta-analysis to investigate the global empathy ability of nursing students, this paper reports that the global empathy ability among nursing students was SMD of 7.99 (95% CI 7.00–8.98) with significant heterogeneity across the studies (χ² = 4.39; *df* = 19, *P* < .00001; *I*² = 95.0%). Among 19 articles, there have been 5 articles^[12–14,16,27]^ with no detailed report empathy ability level, and there have been 3 articles,^[15,18,28]^ 4 articles,^[11,22,25,26]^ and 7 articles^[17,19–21,23,24,29]^ that report on low, medium, and high levels of nursing students’ empathy ability, respectively. It can be seen that most literature reports that the empathy level of nursing students is mainly at a high level. The studies showed that undergraduate nursing students show a significantly higher mean score of empathy than those attending other undergraduate courses.^[30,31]^ Petrucci et al^[14]^ provided that might be explained by students who choose nursing programs may have a particular aptitude for establishing helping relationships with other people, which is a key point of the nursing profession.

### 4.2. Subgroup discussion

A comparison of empathy ability between female and male nursing students has revealed no significant difference (*P* = .16). Females nursing students have a higher empathy ability than males. This result is similar to the studies that examined students’ empathic skill levels based on their genders.^[14]^ Female students have stronger emotional expressions than male students, which increases their level of empathy ability. According to Leppel,^[32]^ student gender is an important independent factor when choosing degree courses: women often choose academic courses that women, such as nursing, traditionally dominated. Nursing educators should focus on cultivating male students’ empathy ability and improving male students’ identification with the nursing profession.^[33]^

Subgroup analysis was conducted between rural and provincial nursing students, and a significant difference was observed (*P* = .04). The empathy ability of rural students is higher than that of provincial students. This finding is consistent with that of Yang et al.^[29]^ Better family economic conditions can ensure that nursing students get all their necessities smoothly while growing up. Such natural acquisition will not make nursing students consider satisfying their own needs by pleasing others. However, nursing students from rural families, because their families cannot fully meet their various needs in growth, may strive for themselves by thinking about others and gaining recognition from others, and they may have a stronger tendency to pay attention to others so that they will have higher empathy ability.^[23]^ To improve the empathy ability of nursing students from different family environments, nursing educators should pay attention to nursing students’ psychological development and develop individualized training programs.

### 4.3. Limitations of this review

There are several limitations of this review. First, due to the limitations of research inclusion, there was no analysis of empathy ability among nursing students from different countries. Second, only English and Chinese papers were included in the review, limiting the inclusion of other languages. Third, the review was based on 7 English and 3 Chinese language databases and did not include gray literature sources. Therefore, the conclusions should be treated with caution.

## 5. Conclusion

This systematic review reported higher empathy ability among nursing students worldwide, but there needs further improvement. The meta-analysis has shown that females have a higher empathy ability than males, and the empathy ability of rural students is higher than that of provincial students. In nursing education, nursing educators should pay more attention to male nursing students and those with a poor family economy, develop individualized empathy ability training programs, cultivate nursing students’ intention to engage in nursing work, and improve their emotional understanding ability. Improving the level of empathy is beneficial to cultivating more and more high-quality nursing workers, building a harmonious nurse–patient relationship, providing better quality nursing services for patients and improving the overall level of nursing.

Future studies should continue to integrate the factors influencing the empathy ability of nursing students, such as the influence of the distribution of countries, family income, parents’ status, and whether to serve as a class cadre.

## Author contributions

JJR: designed the meta-analysis, extracted the data, performed the meta-analysis, wrote the first draft and revise manuscript.

ZYX: designed the meta-analysis, extracted the data, performed the meta-analysis.

HWN: supervision, reviewed the articles.

All authors have read and approved the final draft.
